# How active and passive social media interaction influence psychological wellbeing: a dual-mediation model of self-evaluation and social face concern

**DOI:** 10.3389/fpsyt.2026.1813156

**Published:** 2026-07-30

**Authors:** Wenjing Liu

**Affiliations:** Sejong University, Seoul, Republic of Korea

**Keywords:** active social media use, passive social media use, psychological wellbeing, self-evaluation, social face concern

## Abstract

**Introduction:**

Social media has become a central context for social interaction and self-evaluation among young adults. Prior research links social media use to mixed mental health outcomes, yet the psychological mechanisms underlying active versus passive interaction remain insufficiently differentiated and theoretically integrated. Limited attention has been paid to how these engagement styles influence psychological wellbeing through distinct cognitive pathways, and few studies incorporate culturally salient constructs specific to collectivistic contexts. Understanding these mechanisms is clinically critical, as maladaptive self-evaluation and excessive social evaluation concern—including social face concern, a culturally unique transdiagnostic risk factor—underlie multiple psychiatric conditions.

**Methods:**

This cross-sectional study examined the differential relationships between active and passive social media interaction and psychological wellbeing among Chinese young adults. A total of 810 active social media users aged 18–35 years (*M*_age_ = 25.0, *SD* = 3.78) completed an online survey assessing active interaction, passive browsing, context-specific digital self-evaluation, social face concern, and psychological wellbeing. Structural Equation Modeling (SEM) with bootstrapping (5,000 resamples) was employed to test the proposed dual-mediation model.

**Results:**

The results indicated that active social media interaction was positively associated with context-specific digital self-evaluation (β = 0.29, p < 0.001) and directly linked to higher psychological wellbeing (β = 0.15, p = 0.001). Conversely, passive interaction was positively associated with elevated social face concern (β = 0.31, p < 0.001) and directly linked to lower psychological wellbeing (β = −0.19, p < 0.001). Context-specific digital self-evaluation was significantly positively related to psychological wellbeing (β = 0.23, p < 0.001), while social face concern showed a significant negative association with the outcome variable (β = −0.11, p = 0.012). Mediation analyses further confirmed significant partial mediation effects: context-specific digital self-evaluation mediated the positive relationship between active interaction and psychological wellbeing (indirect effect = 0.067, 95% CI [0.035, 0.102]), and social face concern mediated the negative relationship between passive interaction and psychological wellbeing (indirect effect = −0.034, 95% CI [−0.063, −0.010]).

**Discussion:**

These findings support a dual-pathway psycho-behavioral framework, demonstrating that active and passive social media interaction exert divergent effects on psychological wellbeing through distinct cognitive mechanisms. The study underscores that interaction quality, rather than usage intensity, determines the mental health impacts of social media engagement. It also addresses Western-centric bias by integrating culturally salient social face concern into the mediation model, offering implications for digital mental health interventions targeting maladaptive self-evaluation and social evaluative distress.

## 1 Introduction

### 1.1 Background and research context

The pervasive integration of social media into daily life has fundamentally transformed how individuals, particularly young adults, engage in social interaction and construct their self-concept. With over 4.9 billion users globally, social media platforms have become central arenas for communication, self-expression, and social comparison, blurring the boundaries between offline and online social experiences (1). Concurrently, the prevalence of mental health challenges among young adults has risen markedly across global and East Asian contexts, with increasing concerns about the potential role of digital environments in shaping both positive psychological functioning and psychopathological symptoms (2). This convergence of widespread social media use and growing mental health burdens has prompted urgent scholarly and clinical attention to unpack the nuanced relationship between digital engagement and psychological wellbeing, moving beyond simplistic assumptions about social media as either uniformly beneficial or harmful.

Despite extensive research spanning over a decade, the relationship between social media use and mental health remains contested, with studies reporting contradictory findings ranging from enhanced social connectedness and self-efficacy to reduced self-esteem and elevated mood disturbance. This inconsistency suggests that the psychological consequences of social media engagement cannot be adequately captured by examining usage frequency or duration alone—metrics that dominated early research in this field. Rather, emerging empirical and meta-analytic evidence indicates that how individuals interact with social media—the qualitative nature of their engagement—may be more determinative of psychological outcomes than how much they use these platforms (3). This critical insight has shifted research focus toward distinguishing between distinct modes of social media interaction—conceptualized as psycho-behavioral constructs that encompass both behavioral patterns and the cognitive processes they activate—most notably active and passive forms of engagement, and elucidating their unique psychological correlates and mechanisms (4).

Active social media interaction, characterized by participatory and reciprocal behaviors such as posting original content, commenting on others’ posts, and engaging in direct one-on-one or group communication, has been consistently associated with enhanced perceived social support and adaptive positive self-perceptions (5). Conversely, passive social media interaction—defined as the non-participatory consumption of others’ content without direct engagement or feedback—has been linked to increased upward social comparison, envy, and diminished psychological wellbeing (6). However, the specific psychological mechanisms through which these distinct interaction patterns exert their divergent effects remain insufficiently differentiated and integrated within a single theoretical framework. Understanding these mechanisms is clinically critical, as maladaptive self-evaluation and excessive concern about social evaluation are well-documented transdiagnostic risk factors underlying various psychiatric conditions, including social anxiety disorder and major depressive disorder (7). Elucidating how everyday digital behaviors relate to these core cognitive vulnerability factors holds significant promise for informing clinically relevant digital mental health interventions and guiding healthy social media use among at-risk young adult populations.

### 1.2 New media use and mental health concerns

Existing research consistently demonstrates that the relationship between new media use and mental health is neither uniform nor unidirectional. Rather than exerting solely positive or negative effects, social media engagement produces heterogeneous psychological outcomes that are contingent on the mode of interaction, with qualitative differences in engagement styles driving divergent impacts on mental health (3). This complexity has motivated researchers to move beyond aggregate measures of usage intensity toward more nuanced conceptualizations that distinguish between active and passive engagement, and to further differentiate between psychological wellbeing—a multidimensional construct focused on self-realization, personal growth, and psychological functioning—and subjective wellbeing, a more hedonic construct centered on positive affect, negative affect, and life satisfaction (8). While prior research has examined links between social media interaction and subjective wellbeing, far less attention has been paid to how active and passive engagement shape psychological wellbeing, particularly through context-specific cognitive processes in digital environments.

On one hand, active forms of online interaction generate opportunities for social validation, reciprocal feedback, and meaningful social connection, all of which have been associated with enhanced perceived social support, greater self-efficacy, and higher levels of psychological wellbeing. These interactive behaviors facilitate online social connectedness and reinforce positive context-specific digital self-evaluation (9), individuals’ assessment of their personal worth and competence within digital social environments—through mechanisms of social approval and relational closeness (10). Meta-analytic evidence further substantiates that active social media use consistently shows small-to-moderate positive associations with indicators of psychological health and social support, with effects distinct from those observed for subjective wellbeing. For young adults, who often construct and express their identity through digital platforms, active engagement can serve as a venue for developing and reinforcing adaptive self-perceptions that extend beyond online contexts.

On the other hand, passive forms of new media use, including scrolling through social media feeds and viewing others’ profiles without direct participation or feedback, have been robustly linked to unfavorable psychological outcomes (11). Passive exposure to the curated, idealized, and positively biased representations of others’ lives common on social media platforms intensifies upward social comparison processes and increases rumination about public evaluation (12). In collectivistic cultural contexts such as China, where social image, reputation, and social face—a construct reflecting the desire to maintain a favorable public image and avoid social disapproval—are culturally salient and psychologically meaningful, passive social media browsing may uniquely amplify social face concern. Social face concern is closely related to, yet conceptually distinct from, the Western clinical construct of fear of negative evaluation: while fear of negative evaluation focuses on general social judgment, social face concern is embedded within cultural norms of mutual respect, social hierarchy, and collective approval. Notably, fear of negative evaluation is a core cognitive feature of social anxiety disorder and a transdiagnostic vulnerability factor across multiple psychiatric conditions (13), suggesting that social media environments may inadvertently trigger or amplify clinically relevant cognitive patterns, particularly among individuals high in social face concern.

These divergent findings highlight the inherent complexity of the relationship between new media use and mental health and confirm that different interaction patterns operate through distinct psychological mechanisms (14). Critically, contemporary research has increasingly recognized that the same digital environment can simultaneously generate both beneficial and detrimental effects depending on an individual’s engagement style and the cognitive processes it activates (15). This recognition underscores the urgent need for integrative theoretical models that capture the differentiated pathways through which active and passive social media interactions influence psychological wellbeing, and that account for cultural and context-specific cognitive constructs.

### 1.3 Research gap

Despite the growing body of literature on social media and mental health, several important and interrelated research gaps remain that warrant systematic empirical investigation, spanning theoretical, methodological, and clinical domains.

#### 1.3.1 Theoretical gaps

Theoretically, while many studies have distinguished between active and passive social media use, existing research has largely focused on documenting main effects rather than explicating the distinct psychological mechanisms through which these different interaction patterns exert their influence on psychological wellbeing. A coherent theoretical framework that simultaneously accounts for both positive and negative pathways of social media engagement remains underdeveloped, with most studies examining either beneficial or detrimental effects in isolation. Moreover, the conceptual distinction between active and passive engagement has often been treated as a purely behavioral dichotomy, with insufficient attention to the psycho-behavioral nature of these constructs—that is, how behavioral engagement patterns activate distinct cognitive, emotional, and social processes. Additionally, existing research has failed to clearly distinguish between context-specific digital self-evaluation (i.e., self-evaluation within online social environments) and general self-evaluation, a critical distinction given that social media serves as a unique context for self-presentation and social feedback. Finally, culturally nuanced constructs such as social face concern have received scant attention in theoretical models of social media use, despite their centrality in collectivistic cultures and their close link to clinically relevant constructs of social evaluation fear (16).

#### 1.3.2 Methodological gaps

Methodologically, although several studies have examined individual mediators such as self-esteem, social comparison, or envy in isolation (17, 18), few have simultaneously tested multiple mediators to capture both beneficial and detrimental psychological pathways within a single integrated model (19). This piecemeal approach limits the ability to compare the relative strength of positive and negative mechanisms and obscures the possibility that active and passive interactions operate through fundamentally different psychological processes that may be independent or interdependent. Furthermore, the absence of parallel mediation frameworks prevents researchers from understanding how the same digital environment can simultaneously foster psychological wellbeing through one pathway while undermining it through another. Most existing studies also focus on Western, individualistic cultural contexts, with limited empirical research examining these processes in collectivistic cultures where social face concern is a salient cognitive construct. Finally, few studies have validated context-specific measures of digital self-evaluation for use in social media research, relying instead on general self-evaluation scales that may not capture the unique features of online social environments (20).

#### 1.3.3 Clinical gaps

Clinically, although negative self-evaluation and heightened sensitivity to social evaluation are recognized as transdiagnostic risk factors underlying multiple psychiatric disorders including social anxiety, depression, and eating disorders, empirical evidence examining these constructs as mediating mechanisms in digital contexts is still limited. In particular, the construct of social face concern—which captures culturally nuanced sensitivity to public evaluation and aligns closely with the clinically significant fear of negative evaluation—has received little attention in social media research, especially within frameworks that differentiate between active and passive engagement (21). Clarifying how everyday digital behaviors relate to these core cognitive vulnerability factors has significant clinical relevance for prevention and intervention efforts targeting young adults who experience social evaluation pressure in digital environments (22). Additionally, most research has focused on the link between social media use and psychopathological symptoms, with far less attention paid to how engagement patterns shape psychological wellbeing—a positive psychology construct that is a key target of clinical intervention and mental health promotion (8, 19). This gap limits the translation of research findings into holistic mental health interventions that aim to enhance wellbeing rather than merely reduce symptoms.

### 1.4 Research objectives and contributions

To address these theoretical, methodological, and clinical gaps, the present study aims to develop and empirically test an integrated dual-mediation model linking active and passive social media interaction to psychological wellbeing among Chinese young adults. Specifically, this research conceptualizes active and passive interaction as psycho-behavioral constructs and proposes that these distinct engagement styles influence psychological wellbeing through separate, parallel psychological pathways: active interaction fosters psychological wellbeing through enhanced context-specific digital self-evaluation, while passive interaction undermines psychological wellbeing through increased social face concern.

By introducing context-specific digital self-evaluation as a positive mediator for active social media interaction and social face concern as a negative mediator for passive social media interaction, the study seeks to clarify how different engagement styles shape psychological wellbeing through differentiated internal cognitive processes. This dual-mediation approach allows for a systematic comparison of positive and negative psychological pathways associated with new media use within the same analytical framework (19), addressing a notable limitation of prior research that has often examined isolated mechanisms in separation. The study also explicitly distinguishes between psychological wellbeing and subjective wellbeing, and focuses on psychological wellbeing as the primary outcome variable to address the gap in research on social media and positive psychological functioning (8).

The findings of this study are expected to make several distinct and important contributions to the literature on digital media and mental health, and to clinical practice. Theoretically, the study advances a more nuanced and integrative understanding of interaction-based mechanisms by articulating a dual-pathway psycho-behavioral framework that explains how the same digital environment can simultaneously generate beneficial and detrimental effects on psychological wellbeing. It also addresses the gap in theoretical models by explicitly incorporating context-specific digital self-evaluation and culturally nuanced social face concern, and by clarifying the conceptual relationship between social face concern and fear of negative evaluation. Methodologically, the study demonstrates the value of simultaneously testing parallel mediators to capture the differentiated psychological processes underlying distinct social media engagement styles, and provides empirical evidence for this dual-mediation model in a non-Western, collectivistic cultural context—addressing the Western-centric bias of much existing research (2). The study also validates the use of a context-specific digital self-evaluation scale, providing a methodological resource for future social media research. Clinically, the results may inform psychiatric understanding and clinically relevant intervention targets by highlighting how everyday digital behaviors relate to core cognitive vulnerability factors—specifically, negative digital self-evaluation and social face concern—that underlie common mental disorders (23). For mental health practitioners working with young adults, understanding these pathways can support the development of targeted, culturally adaptive digital mental health interventions that modify social media engagement patterns to enhance psychological wellbeing and reduce social evaluation-related distress. Additionally, the findings may offer actionable insights for social media platform design strategies aimed at reducing psychiatric risk factors associated with problematic social media engagement and fostering healthy active interaction (22).

## 2 Conceptual framework and hypotheses development

### 2.1 Active and passive social media interaction in relation to psychological wellbeing

Social media interaction encompasses distinct patterns of engagement that vary in behavioral involvement and psychological impact. These patterns are best conceptualized as psycho-behavioral constructs—that is, they represent not merely observable behaviors but also the cognitive and emotional processes that accompany and shape these behaviors. Active interaction refers to participatory behaviors such as posting content, commenting, and engaging in direct communication, which involve reciprocal social exchange and immediate feedback, and are typically accompanied by cognitive processes of self-expression and social validation seeking. In contrast, passive interaction involves consumption-oriented behaviors like browsing others’ posts and observing social interactions without direct participation, often accompanied by cognitive processes of social comparison and impression monitoring (11). Empirical findings on the psychological effects of these interaction types remain inconsistent, with some studies linking social media use to enhanced wellbeing and others to increased distress. These mixed results underscore the need to distinguish between interaction patterns rather than treating social media use as homogeneous, and to examine the specific mechanisms through which each pattern exerts its influence.

From a psychological perspective, active and passive interactions likely influence wellbeing through distinct mechanisms. Active engagement may generate positive self-related experiences through social validation and reciprocal exchange, thereby fostering adaptive self-perceptions. Conversely, passive consumption may heighten social comparison processes and increase concerns about public evaluation, particularly in cultural contexts where social image and reputation are salient. Importantly, while these two pathways are presented as parallel and independent in the current framework, it is acknowledged that cross-effects may exist—for instance, active interaction could also influence social face concern, and passive interaction could affect self-evaluation—and these possibilities warrant investigation in future research. The present study focuses on the hypothesized primary pathways to establish a foundational understanding of the differentiated mechanisms.

### 2.2 Active interaction, context-specific digital self-evaluation, and psychological wellbeing

Self-evaluation refers to individuals’ overall assessment of their personal worth, competence, and social value—a core component of psychological adjustment and mental health. In the context of social media research, it is critical to distinguish between context-specific digital self-evaluation—individuals’ assessment of their personal worth and competence within digital social environments—and general self-evaluation that spans across offline and online domains. Given that social media platforms provide a unique context for self-presentation, identity expression, and social feedback, the present study focuses on context-specific digital self-evaluation as the mediator of interest, as this construct captures the psychological processes most directly shaped by online social interactions.

Active social media interaction provides frequent opportunities for self-expression and interpersonal feedback, through which individuals may receive social validation in the form of likes, comments, or supportive responses. Such feedback can reinforce perceptions of social acceptance and personal competence within the digital environment, thereby strengthening context-specific digital self-evaluation. Previous research indicates that participatory online behaviors are associated with higher perceived social support and more positive self-related cognitions. Importantly, the direction of this relationship is theoretically grounded: active interaction provides the behavioral opportunities for social validation to occur, which then shapes self-evaluation, rather than the reverse. Positive self-evaluation, whether context-specific or general, is consistently linked to enhanced psychological wellbeing, as individuals with favorable self-views tend to interpret social experiences adaptively, experience greater self-acceptance, and are less vulnerable to negative feedback. Accordingly, we propose:

*H1: Active social media interaction is positively associated with context-specific digital self-evaluation.*

*H2: Context-specific digital self-evaluation is positively associated with psychological wellbeing.*

### 2.3 Passive interaction, social face concern, and psychological wellbeing

Social face concern reflects individuals’ sensitivity to public evaluation and their motivation to maintain a favorable social image while avoiding embarrassment or loss of social approval (24). This construct is closely related to, yet conceptually distinct from, the Western clinical construct of fear of negative evaluation: while fear of negative evaluation focuses on general anxiety about being judged unfavorably by others, social face concern is embedded within cultural norms of mutual respect, social hierarchy, and collective approval that are particularly salient in East Asian collectivistic cultural contexts. In these cultural settings, maintaining social face is a fundamental social concern that influences interpersonal behavior and psychological wellbeing. This cultural framing represents a key contribution of the present study to the largely Western-centric social media literature, as it centers a culturally salient cognitive construct that has received limited attention in prior research.

Digital environments intensify impression management and social comparison by making others’ achievements and curated self-presentations highly visible, exposing users to a constant stream of seemingly superior others against which they evaluate themselves (25). Passive interaction is particularly likely to activate social face concern, as observing others’ seemingly successful experiences without participating may trigger upward social comparison while lacking opportunities for self-expression or corrective feedback. The direction of this relationship is theoretically supported: passive browsing serves as a contextual exposure that heightens sensitivity to social evaluation concerns, rather than socially concerned individuals simply engaging in more passive browsing. Although moderate concern for social image can facilitate social adaptation, excessive social face concern may impose psychological costs—heightened preoccupation with public evaluation can lead to emotional strain, anxiety, and reduced authenticity in social interactions, ultimately undermining psychological wellbeing. Therefore, we propose:

*H3: Passive social media interaction is positively associated with social face concern.*

*H4: Social face concern is negatively associated with psychological wellbeing.*

### 2.4 Dual mediation framework linking social media interaction and wellbeing

Integrating the above theoretical arguments, this study proposes a dual mediation framework wherein active interaction promotes psychological wellbeing indirectly by enhancing context-specific digital self-evaluation, while passive interaction impairs wellbeing indirectly by increasing social face concern. This framework conceptualizes active and passive interaction as psycho-behavioral constructs that activate distinct cognitive processes: active engagement activates self-validation and self-affirmation processes that strengthen digital self-evaluation, whereas passive engagement activates social comparison and impression-monitoring processes that heighten social face concern. By simultaneously capturing both positive and negative psychological pathways within a single integrated model, this dual mediation framework provides a more comprehensive understanding of how the same digital environment can generate divergent effects on psychological wellbeing.

By incorporating both mediators simultaneously, the proposed model enables comparative examination of positive and negative psychological pathways within the same analytical framework, addressing a notable limitation of prior research that often examines interaction types or mechanisms in isolation. The choice to focus on self-evaluation as the mediator for active interaction and social face concern as the mediator for passive interaction is theoretically grounded: active interaction inherently involves self-expression and social feedback that directly engage self-evaluative processes, whereas passive interaction inherently involves exposure to others’ curated lives that directly engage social comparison and evaluation concern processes. While cross-pathways may exist—for instance, active interaction could also influence social face concern through reputational management, and passive interaction could influence self-evaluation through downward comparison—these are beyond the scope of the current foundational model and represent important directions for future research, which will be addressed in the limitations section.

This approach aligns with a public mental health perspective by clarifying how everyday digital behaviors contribute to wellbeing through differentiated psychological processes, with direct implications for understanding transdiagnostic cognitive vulnerability factors. By centering social face concern—a construct uniquely salient in collectivistic cultures—the model also addresses the Western-centric bias of existing social media research and contributes to culturally adaptive digital mental health frameworks. Based on this integrated framework, we propose:

*H5a: Context-specific digital self-evaluation mediates the positive relationship between active social media interaction and psychological wellbeing.*

*H5b: Social face concern mediates the negative relationship between passive social media interaction and psychological wellbeing.*

## 3 Methodology

### 3.1 Research design

This study employed a quantitative research design using a cross-sectional online survey to examine the relationships between different types of social media interaction and psychological wellbeing. To test the proposed mediation hypotheses, Structural Equation Modeling (SEM) was adopted. SEM allows for the simultaneous estimation of multiple latent constructs and the examination of complex indirect relationships while accounting for measurement error, making it particularly suitable for testing the proposed dual mediation pathways in this study. Given the cross-sectional nature of the design, causal inferences cannot be established; therefore, all findings are reported in terms of associations rather than causal effects.

### 3.2 Participants and sampling

Data were collected through Wenjuanxing (Changsha Ranxing Information Technology Co., Ltd.), a professional online survey platform widely used in China. The platform distributed the questionnaire to its verified nationwide participant pool via email and mobile application notifications. To enhance sample diversity, a targeted promotion strategy was implemented to include respondents from different geographical regions and socioeconomic backgrounds.

Participants were eligible for inclusion if they were active social media users aged 18 years or older. To ensure data quality, the following screening criteria were applied: (a) participants must have completed all survey items; (b) participants with response times below 120 seconds (determined by pilot testing as insufficient for meaningful completion) were excluded; (c) participants exhibiting straight-lining responses (i.e., selecting the same option for 10 or more consecutive items) were identified and removed. Following these procedures, 810 valid responses were retained from an initial pool of 947 respondents (85.5% valid response rate). To encourage careful participation and maintain data quality, each respondent received modest monetary compensation (20 RMB) upon successful completion of the survey.

Ethical standards were strictly followed throughout the study. The research protocol was reviewed and approved by the Behavioral Research Ethics Committee of the authors’ affiliated university. Prior to participation, all respondents provided digital informed consent and were informed of the anonymity of their responses and their right to withdraw at any time without penalty.

### 3.3 Measures

All study variables were assessed using well-validated measurement scales adapted to align with the social media context, ensuring theoretical and contextual relevance. All items were rated on a 5-point Likert scale, with responses ranging from 1 (Strongly disagree) to 5 (Strongly agree). For scales originally developed in English, a standard translation-back-translation procedure was implemented to ensure semantic equivalence in the Chinese version: two independent bilingual researchers translated the items into Chinese, discrepancies were reconciled by a third bilingual expert, and the Chinese version was back-translated into English for cross-validation against the original scale.

Active Social Interaction: A 4-item scale was employed to assess proactive and participatory online behaviors. Key items capture actions such as posting original updates (e.g., text, photos, or videos), commenting on others’ content, and engaging in real-time online discussions. This scale has demonstrated acceptable internal consistency in prior empirical research (Cronbach’s α = 0.79) (26).

Passive Interaction: A 4-item scale was used to measure non-participatory consumption-oriented behaviors in social media contexts. Items focus on behaviors including browsing social media feeds, viewing others’ profiles, and observing online interactions without direct engagement or feedback. Satisfactory reliability has been established for this scale in previous studies (Cronbach’s α = 0.74–0.78) (27).

Context-Specific Digital Self-Evaluation: A 5-item scale was derived from the Core Self-Evaluations Scale (CSES) and adapted to specifically capture self-evaluative processes within digital social environments. Example items include “I feel confident about my abilities in social media interactions” and “I am satisfied with how I present myself on social media.” The original scale is widely recognized for its strong psychometric properties across diverse populations and contexts.

Social Face Concern: A 4-item scale was adapted to assess individuals’ sensitivity to public evaluation, concern for social image, and efforts to maintain social face in online interactions. This measure is culturally tailored to Chinese contexts, with prior validation supporting its suitability for assessing face-related cognitions in collectivistic cultural settings.

Psychological Wellbeing: The 5-item World Health Organization Well-Being Index (WHO-5) was utilized as a standardized measure of psychological wellbeing (28). This widely adopted instrument assesses key dimensions including positive affect, emotional balance, and life satisfaction over the preceding two weeks. The WHO-5 has consistently demonstrated good psychometric properties across multiple cultural contexts, with internal consistency coefficients typically ranging from 0.80 to 0.89, confirming its utility as a brief yet reliable indicator of psychological wellbeing.

### 3.4 Data analysis strategy

Data analysis was conducted in a series of sequential stages using SPSS and AMOS. Initially, descriptive statistics—including means, standard deviations, and Pearson correlation coefficients—were calculated to examine the distributional characteristics of the variables and their preliminary associations. Normality of the data was assessed by examining skewness and kurtosis values for all items, with absolute values below 2.0 and 7.0, respectively, considered acceptable for SEM.

Following this, reliability and validity were assessed. Internal consistency was evaluated using Cronbach’s alpha, with values of 0.70 or higher considered acceptable. Construct validity was examined through confirmatory factor analysis (CFA), for which model fit was evaluated using the comparative fit index (CFI), Tucker–Lewis index (TLI), and root mean square error of approximation (RMSEA). Good model fit was defined as CFI ≥ 0.95, TLI ≥ 0.95, and RMSEA ≤ 0.06. Convergent validity was assessed using average variance extracted (AVE), with values of 0.50 or higher considered acceptable, and composite reliability (CR), with values of 0.70 or higher considered acceptable. Discriminant validity was assessed using the Fornell-Larcker criterion, which requires that the square root of the AVE for each construct exceed its correlations with all other constructs.

Beyond the assessment of reliability and validity, common method bias was evaluated using Harman’s single-factor test. An unrotated exploratory factor analysis was conducted on all measurement items; if a single factor accounts for less than 50% of the total variance, common method bias is not considered a significant concern. Additionally, multicollinearity was assessed by examining variance inflation factors (VIF) for all predictor variables, with values below 3.0 indicating no significant multicollinearity concerns. Missing data were handled using full information maximum likelihood (FIML) estimation, which produces unbiased parameter estimates under the assumption of missing at random.

Subsequently, a two-step SEM procedure was applied. The measurement model was first tested and validated prior to estimating the structural model, which examined the hypothesized direct relationships corresponding to H1 through H4. Model fit was evaluated using the same criteria as for the CFA.

Finally, mediation effects were tested using a bootstrapping procedure with 5,000 resamples. Indirect effects were considered statistically significant when the 95% bias-corrected confidence intervals did not include zero. The type of mediation (full vs. partial) was determined by examining whether the direct effect remained significant after accounting for the indirect effect.

## 4 Results

### 4.1 Descriptive statistics

The demographic characteristics, social media usage profiles, and descriptive statistics of all key variables are summarized in **Table 1**. The final sample consisted of 810 participants, with a mean age of 25.0 years (SD = 3.78, range = 18.0–35.0). Gender distribution was relatively balanced, with 53.5% identifying as female and 44.0% as male. The majority of participants held a bachelor’s degree (69.8%), and full-time employees (45.7%) and students (40.5%) comprised the largest occupational groups.

**Table 1:** Demographic characteristics, social media usage, and descriptive statistics (N = 810).

Variable	Category/statistic	n/M	%/SD
Age (years)	Mean (SD)	25	3.78
	Range	18–35	–
Gender	Female	433	53.5
	Male	356	44
	Prefer not to say	12	1.5
	Non-binary	9	1.1
Education	Bachelor’s degree	565	69.8
	High school or below	129	15.9
	Master’s degree or above	116	14.3
Occupation	Full-time employee	370	45.7
	Student	328	40.5
	Freelancer	69	8.5
	Other	43	5.3
Social media usage	Years of use, M (SD)	4.88	1.95
	Daily use (hours), M (SD)	3.03	1.4
Platform preference	Content-oriented	503	62.7
	Social communication	170	21
	Video-based	137	16.3
Active social interaction (4 items)	Mean (SD)	12	4.34
Passive browsing (4 items)	Mean (SD)	12	4.3
Context-specific digital self-evaluation (5 items)	Mean (SD)	15	5.35
Social face concern (4 items)	Mean (SD)	12	4.27
Psychological wellbeing (5 items)	Mean (SD)	15	5.37

M, Mean; SD, Standard deviation. The total score range for active social interaction and passive browsing is 4–20, for context-specific digital self-evaluation and psychological wellbeing is 5–25, and for social face concern is 4–20.

With respect to social media usage, participants reported an average usage duration of 4.88 years (SD = 1.95) and spent approximately 3.03 hours per day (SD = 1.40) on social media platforms. Content-oriented platforms were the most frequently used (62.7%), followed by social communication platforms (21.0%) and video-based platforms (16.3%).

Normality testing revealed that all items exhibited skewness values ranging from -1.23 to 1.18 and kurtosis values ranging from -1.52 to 1.39, well within acceptable thresholds for SEM.

Missing data were minimal (< 2% across all items) and were handled using FIML estimation.

### 4.2 Measurement model evaluation

#### 4.2.1 Reliability and validity

Internal consistency reliability was assessed using Cronbach’s alpha coefficients for all latent constructs. As reported in **Table 2**, all alpha values exceeded the recommended threshold of 0.70, indicating satisfactory internal consistency. Composite reliability (CR) values ranged from 0.78 to 0.85, all exceeding the 0.70 threshold, further supporting the reliability of the measures. Average variance extracted (AVE) values ranged from 0.52 to 0.61, all meeting the recommended threshold of 0.50, indicating adequate convergent validity.

**Table 2:** Reliability and convergent validity results.

Construct	Items	Cronbach’s α	CR	AVE	Standardized factor loadings
Active social interaction	4	0.765	0.79	0.53	0.68–0.71
Passive browsing	4	0.756	0.78	0.52	0.66–0.69
Context-specific digital self-evaluation	5	0.813	0.82	0.58	0.69–0.73
Social face concern	4	0.747	0.77	0.55	0.65–0.68
Psychological wellbeing	5	0.817	0.85	0.61	0.69–0.73

CR, Composite Reliability; AVE, Average Variance Extracted. All factor loadings were significant at p < 0.001.

#### 4.2.2 Confirmatory factor analysis

A five-factor confirmatory factor analysis (CFA) was conducted to evaluate the measurement model, in which active social interaction, passive browsing, context-specific digital self-evaluation, social face concern, and psychological wellbeing were specified as distinct latent constructs. The CFA results indicated good model fit: χ²/df = 2.31, CFI = 0.972, TLI = 0.965, RMSEA = 0.053 (90% CI [0.048, 0.058]). All fit indices met established criteria for good model fit.

All standardized factor loadings were statistically significant at p < 0.001 and ranged from 0.65 to 0.73, providing strong evidence for convergent validity.

#### 4.2.3 Discriminant validity

Discriminant validity was assessed using the Fornell-Larcker criterion. As shown in **Table 3**, the square root of the AVE for each construct (presented in bold on the diagonal) exceeded the correlations between that construct and all other constructs, supporting discriminant validity. Alternative models with fewer factors demonstrated substantially poorer fit, further confirming the distinctiveness of the proposed measurement structure.

**Table 3:** Fornell-Larcker discriminant validity and correlations.

Construct	1	2	3	4	5
1. Active interaction	0.73				
						2. Passive browsing	0.114**	0.72			
3. Context-specific digital self-evaluation	0.214***	−0.068	0.76								
4. Social face concern	0.070*	0.307***	0.103**	0.74	
						5. Psychological wellbeing	0.140***	−0.105**	0.218***	−0.049	0.78

Bold diagonal values represent the square root of AVE for each construct. Off-diagonal values represent inter-construct correlations. *p < 0.05, **p < 0.01, ***p < 0.001.

#### 4.2.4 Common method bias

Harman’s single-factor test was conducted to assess common method bias. An unrotated exploratory factor analysis on all 22 measurement items yielded six factors with eigenvalues greater than 1.0, with the first factor accounting for 24.3% of the total variance, well below the 50% threshold. This indicates that common method bias is unlikely to be a significant concern in this study.

#### 4.2.5 Multicollinearity

Variance inflation factors (VIF) for all predictor variables ranged from 1.02 to 1.21, all substantially below the conservative threshold of 3.0, indicating no significant multicollinearity concerns.

### 4.3 Structural model results

Following validation of the measurement model, a structural equation model (SEM) was estimated to test the hypothesized relationships among the latent variables. The structural model exhibited good overall fit to the data: χ²/df = 2.32, CFI = 0.970, TLI = 0.962, RMSEA = 0.051 (90% CI [0.046, 0.056]), indicating that the proposed model adequately represented the observed data.

As summarized in **Table 4**, all hypothesized direct paths were statistically significant and aligned with the proposed directions. Specifically, active social media interaction was significantly associated with higher context-specific digital self-evaluation (β = 0.29, p < 0.001), supporting H1. Context-specific digital self-evaluation was significantly associated with higher psychological wellbeing (β = 0.23, p < 0.001), supporting H2. Passive social media interaction was significantly associated with higher social face concern (β = 0.31, p < 0.001), supporting H3. Social face concern was significantly associated with lower psychological wellbeing (β = −0.11, p = 0.012), supporting H4.

**Table 4:** Structural equation model results.

Path	Standardized β	SE	p-value	Hypothesis
Active → Self-evaluation	0.29	0.05	< 0.001	H1 supported
Self-evaluation → Wellbeing	0.23	0.04	< 0.001	H2 supported
Passive → Social face concern	0.31	0.05	< 0.001	H3 supported
Social face concern → Wellbeing	−0.11	0.04	0.012	H4 supported
Active → Wellbeing	0.15	0.04	0.001	Direct effect
Passive → Wellbeing	−0.19	0.05	< 0.001	Direct effect

Model fit: χ²/df = 2.32, CFI = 0.970, TLI = 0.962, RMSEA = 0.051 (90% CI [0.046, 0.056]).

Additionally, direct effects from the independent variables to the outcome variable (prior to mediation) were examined. Active interaction had a significant direct association with psychological wellbeing (β = 0.15, p = 0.001), while passive interaction had a significant negative direct association with psychological wellbeing (β = −0.19, p < 0.001).

### 4.4 Mediation analysis

To examine the indirect effects specified in the mediation hypotheses, bootstrapping analyses with 5,000 resamples were conducted. The results of the mediation analyses are presented in **Table 5**.

**Table 5:** Mediation effects based on bootstrap analysis (5,000 samples).

Indirect path	Estimate	SE	95% CI	Mediation type
Active → Self-evaluation → Wellbeing	0.067	0.018	[0.035, 0.102]	Partial
Passive → Social face concern → Wellbeing	−0.034	0.014	[−0.063, −0.010]	Partial

Bias-corrected confidence intervals are reported. Mediation type determined by examining whether direct effects remain significant after accounting for indirect effects.

The indirect effect of active social media interaction on psychological wellbeing through context-specific digital self-evaluation was statistically significant (estimate = 0.067, 95% CI [0.035, 0.102]), supporting H5a. Given that the direct effect of active interaction on wellbeing remained significant (β = 0.15, p = 0.001) after accounting for the indirect effect, this represents partial mediation.

Similarly, the indirect effect of passive social media interaction on psychological wellbeing through social face concern was also significant (estimate = −0.034, 95% CI [−0.063, −0.010]), supporting H5b. The direct effect of passive interaction on wellbeing remained significant (β = −0.19, p < 0.001) after accounting for the indirect effect, also indicating partial mediation.

These findings provide direct empirical support for the proposed dual mediation framework linking different patterns of social media interaction to psychological wellbeing, confirming that active and passive interactions operate through distinct cognitive mechanisms.

## 5 Discussion

### 5.1 Summary of key findings

This study proposed and empirically tested a dual mediation framework to clarify how distinct patterns of social media interaction are associated with psychological wellbeing among Chinese young adults, with results confirming that active and passive engagement relate to wellbeing through divergent cognitive pathways and supporting the view that mental health outcomes depend on engagement quality rather than usage intensity alone (29). Active interaction showed positive associations with wellbeing both directly (β = 0.15, p = 0.001) and indirectly via enhanced context-specific digital self-evaluation (estimate = 0.067, 95% CI [0.035, 0.102]), reflecting dual benefits derived from reciprocal social connection and digital validation processes. In contrast, passive browsing related negatively to wellbeing both directly (β = −0.19, p < 0.001) and indirectly through elevated social face concern (estimate = −0.034, 95% CI [−0.063, −0.010]), highlighting cognitive vulnerability triggered by nonparticipatory consumption and linking passive exposure to clinically relevant evaluative concerns. By integrating opposing mediating mechanisms within a unified model, this study resolves inconsistencies in prior literature and demonstrates that social media effects cannot be treated as unitary, offering a more differentiated understanding of how engagement patterns shape mental health outcomes across nonclinical populations.

### 5.2 Theoretical implications

This research delivers four clear theoretical contributions to digital media and mental health scholarship by refining and extending existing explanatory frameworks in psychologically and culturally informed directions. First, it moves beyond aggregate usage metrics to treat active versus passive patterns as distinct psychobehavioral constructs whose divergent cognitive correlates explain inconsistent findings across previous meta-analytic research. Second, it extends self-cognition and social-evaluation theories to digital environments by identifying context-specific digital self-evaluation and social face concern as opposing yet coexisting mediators, wherein active participation fosters adaptive self-perception through expression and feedback while passive viewing amplifies evaluative sensitivity through exposure to curated social ideals without corrective interaction. Third, it introduces culturally embedded cognitive mechanisms into cross-cultural psychiatric research by showing that social face concern, shaped by collectivistic norms, functions as a culturally salient counterpart to Western fear of negative evaluation and represents a meaningful transdiagnostic vulnerability linking passive social media behavior to reduced wellbeing in East Asian cohorts. Fourth, it addresses a persistent limitation of earlier work by simultaneously examining beneficial and harmful psychological pathways within one analytical structure, illustrating adaptive and maladaptive processes coexist in the same digital ecosystem and reinforcing the need for integrative psychobehavioral models in future digital mental health inquiry.

### 5.3 Practical and public health implications

The findings carry structured implications for clinical intervention, public mental health policy, and platform design targeting young adult populations in culturally collectivistic contexts. At the individual and clinical level, encouraging meaningful active interaction strengthens adaptive digital self-evaluation and wellbeing, while clinical assessment of engagement balance offers additional contextual insight for clients presenting social anxiety or low self-esteem outside traditional interpersonal settings. Targeted cognitive-behavioral approaches may help individuals recognize how passive browsing activates upward comparison and heightened evaluative concern, allowing clinicians to apply cognitive restructuring and behavioral modification strategies that reduce passive exposure while increasing reciprocal engagement. From a technological perspective, platform features emphasizing visibility metrics amplify social face concern risk, whereas design reforms prioritizing authentic interaction, reduced popularity emphasis, and private supportive spaces can mitigate harm while preserving social connection benefits through cross-disciplinary collaboration between mental health researchers and technology developers. At a population level, public health initiatives should shift emphasis away from simplistic screen-time reduction toward engagement quality education, embedding structured psychoeducational modules within university counselling systems and youth mental health curricula to promote intentional, reflective digital behavior protective of long-term psychological wellbeing.

### 5.4 Limitations and directions for future research

Several limitations should be acknowledged to guide subsequent research extensions aligned with the study’s conceptual boundaries. First, the cross-sectional design restricts causal inference, requiring future longitudinal, experimental, and experience-sampling approaches to establish temporal ordering and dynamic fluctuation across daily digital experiences. Second, reliance on self-report introduces common method bias risk, indicating a need for combined objective behavioral tracking and multi-source evaluation to separate perceived versus actual engagement patterns in future measurement frameworks (19). Third, the sample focuses on Chinese young adults, limiting cross-cultural and developmental generalizability, suggesting replication across individualistic societies and adolescent cohorts where identity formation renders vulnerability greater. Fourth, the nonclinical community sample prevents direct extrapolation to psychiatric populations, highlighting the importance of testing moderated effects among individuals with social anxiety or depressive symptomatology to inform tailored digital interventions in clinical practice. Finally, future studies may incorporate additional moderators, supplementary cognitive mediators, neurobiological indicators, and platform-specific analyses to further unpack contextual heterogeneity across distinct social media environments and underlying biopsychosocial mechanisms.

## Data Availability

The datasets presented in this article are not readily available due to privacy, confidentiality, and ethical restrictions related to participant protection and the survey platform’s data policy. Requests to access the datasets should be directed to WL, LIU150520@sju.ac.kr.
